# Mindin serves as a tumour suppressor gene during colon cancer progression through MAPK/ERK signalling pathway in mice

**DOI:** 10.1111/jcmm.15332

**Published:** 2020-07-02

**Authors:** Xiao‐Shen Cheng, Ya‐Ni Huo, Yan‐Yun Fan, Chuan‐Xing Xiao, Xiao‐Mei Ouyang, Lai‐Ying Liang, Ying Lin, Jian‐Feng Wu, Jian‐Lin Ren, Bayasi Guleng

**Affiliations:** ^1^ Department of Gastroenterology Zhongshan Hospital Affiliated to Xiamen University Xiamen China; ^2^ School of Life Sciences Xiamen University Xiamen China; ^3^ School of Medicine Cancer Research Center & Institute of Microbial Ecology Xiamen University Xiamen China; ^4^ State Key Laboratory of Cellular Stress Biology Xiamen University Xiamen China

**Keywords:** colorectal cancer, MAPK/ERK, mindin

## Abstract

Mindin is important in broad spectrum of immune responses. On the other hand, we previously reported that mindin attenuated human colon cancer development by blocking angiogenesis through Egr‐1–mediated regulation. However, the mice original mindin directly suppressed the syngenic colorectal cancer (CRC) growth in our recent study and we aimed to further define the role of mindin during CRC development in mice. We established the mouse syngeneic CRC CMT93 and CT26 WT cell lines with stable mindin knock‐down or overexpression. These cells were also subcutaneously injected into C57BL/6 and BALB/c mice as well as established a colitis‐associated colorectal cancer (CAC) mouse model treated with lentiviral‐based overexpression and knocked‐down of mindin. Furthermore, we generated mindin knockout mice using a CRISPR‐Cas9 system with CAC model. Our data showed that overexpression of mindin suppressed cell proliferation in both of CMT93 and CT26 WT colon cancer cell lines, while the silencing of mindin promoted in vitro cell proliferation via the ERK and c‐Fos pathways and cell cycle control. Moreover, the overexpression of mindin significantly suppressed in vivo tumour growth in both the subcutaneous transplantation and the AOM/DSS‐induced CAC models. Consistently, the silencing of mindin reversed these in vivo observations. Expectedly, the tumour growth was promoted in the CAC model on mindin‐deficient mice. Thus, mindin plays a direct tumour suppressive function during colon cancer progression and suggesting that mindin might be exploited as a therapeutic target for CRC.

## 1 INTRODUCTION

Colorectal cancer (CRC) is one of the most common cancers.^1^ Up to 90% of patients can be cured with surgery if the disease is diagnosed at an early stage. Therefore, the identification of biomarkers to promote early diagnosis is fundamentally important.^2^ Recently, the cellular and molecular mechanisms underlying the process of colorectal carcinogenesis are markedly understood. Many molecular disorders have been reported in CRC, including mutations of oncogenes (k‐ras, etc), inactivation of tumour suppressor genes (APC, p53 and DCC, etc) and DNA mismatch repair genes (MLH1 and MSH2, etc), disturbances in DNA methylation, microsatellite instability and stem cell proliferation and the involvement of non‐coding RNAs.^3^, ^4^, ^5^, ^6^

The mechanism of CRC initiation has been studied in detail. However, solid tumours are not a clone of cancer cells and composed of multiple cell types and extracellular matrix (ECM).^7^ The ECM, one component of the tumour microenvironment, contains various peptide factors and metabolite that are responsible for transducting environmental signals to cells, and these signals essentially affect all behaviours of cell, including cell proliferation, differentiation and death.^8^, ^9^, ^10^ However, the identity of the ECM protein that is involved in CRC development and progression is not yet completely clear.

Mindin is secreted ECM protein and a member of the mindin‐F‐Spondin family.^11^, ^12^, ^13^ Mindin recognizes LPS through its thrombospondin type 1 repeat domain.^14^ Mindin‐deficient mice exhibit defective clearance of influenza virus followed by the impaired activation of macrophages.^15^ Mindin also serves as an opsonin in the phagocytosis of bacteria.^16^ Mindin might be a double‐edged sword in the CRC and IBD. Mindin was reported as a pattern recognition molecule regards to microbial pathogens and plays an integrin ligand during inflammatory cell recruitment and T cell activation.^16^, ^17^, ^18^ And mindin could promote macrophage phagocytosis through Syk activation and NF‐κB p65 translocation.^19^ What's more, our previous results showed that the mRNA expression of mindin is up‐regulated during dextran sulphate sodium (DSS)‐induced acute intestinal inflammation.^20^ Thus, mindin may play an important role in the inflammatory bowel disease. Mindin plays an important role in metabolic diseases, since mindin can mitigate hepatic steatosis, insulin resistance, obesity and ischemia/reperfusion injury.^21^, ^22^, ^23^ And, mindin induces cell motility and CRC metastasis.^24^ Meanwhile, mindin plays a critical role in the immune responses to tumour cell growth and migration in hepatocellular carcinoma.^25^ Furthermore, our recent studies suggested that the serum level of mindin may be a new biomarker for early detection of CRC.^26^ Mindin was also shown to promote the outgrowth of hippocampal embryonic neurons and inhibited the angiogenesis.^27^ Moreover, mindin reported to be an important regulator of Rho GTPase expression and signalling through the integrin‐mediated activation of Rho GTPases.^17^

As knowledge from the literature, Sirtuin 6 (SIRT6) is a histone deacetylase dependent on nicotinamide adenine dinucleotide (NAD)^+^.^28^ In rodents, SIRT6 acts as a longevity protein.^29^, ^30^ Differently, SIRT6 deficiency caused the developmental retardation in cynomolgus monkeys.^31^ This similar mechanical difference was occurred on mindin gene. In this study, mindin acts as a direct tumour suppressor gene in CRC model of mouse through MAPK/ERK signalling pathway. However, we previously determined that mindin attenuates CRC progression by blocking angiogenesis through Egr‐1–mediated regulation in human cell lines.^26^ Alignment analyses in EBI database (https://www.ebi.ac.uk/Tools/psa/) suggested that the Spondin 2 (mindin) amino acid sequences from Homo sapiens and Mus musculus contain 299/332 (90.1%) in similarity and 279/332 (84.0%) in identity. The difference in amino acid sequences from Homo sapiens and Mus musculus may induce the structural change result in the different mechanisms of mindin attenuating the CRC progression.

## 2 MATERIALS AND METHODS

### 2.1 Ethics statement

The Ethics Committee (No: 20081009) of Zhongshan Hospital affiliated to Xiamen University approved this study. The tumour tissue samples were collected from this hospital. We obtained the written consent from all participants who were involved in the study. All protocols of animal experiments were approved by the Committee for Animal Research of Xiamen University.

### 2.2 Generation of mindin knockout mice using a CRISPR‐Cas9 system

We designed the first exon gRNA sequence for mindin based on the online software developed by Professor Zhang Feng at Massachusetts Institute of Technology. The corresponding gRNA plasmids, which were constructed using T4 DNA ligase (Takara, Dalian, China) and the CRISPR plasmid, were transferred into murine cells (L929). The most efficient gRNA sequences were screened and transferred to a plasmid vector expressing a T7 promoter. The CRISPR plasmid and the gRNA plasmid with the T7 promoter were digested and recovered by phenol/chloroform extraction and were resuspended in nuclease‐free water. The transcribed RNAs, which were products of CRISPR and gRNA plasmids that were transcribed using an Sp6 mMESSAGE mMACHINE Kit (Ambion, Carlsbad, CA), were diluted at a concentration of 20 or 5 ng/µL for CRISPR and gRNA, respectively, and were injected into fertilized eggs of C57BL/6J mice using an injection needle. After injection, the fertilized eggs were returned to surrogate mothers. The following primers were applied with mice genotyping: (forward) 5′‐ATACCCTCTCCCAGGCTAGC‐3′ and (reverse) 5′‐CTTTGCTGAGCGTGGTGAGG‐3′, and data did not show the genotyping.

### 2.3 Cell culture

The CT26 and CMT93 WT cell lines (ATCC, Manassas, VA) were grown in RPMI 1640 and DMEM medium with 10% FBS (Life Technologies, Grand Island, NY) and 1% penicillin G/streptomycin and incubated at 37°C with 95% air and 5% CO_2_.

### 2.4 Establishment of stable mindin knock‐down and overexpression cells

Briefly, the shRNA sequence (5′‐GCGGAAGAATGTATGTAAG‐3′) for the mindin was selected as most efficient sequence by our algorithm. The transfection of the mindin‐targeting shRNA plasmid into the CMT93 and CT26 WT cell lines was performed with Lipofectamine 2000 (Invitrogen, Carlsbad, CA), and the empty PU6 vector was used as controls (PU6). After 24 h of culture, the cells were selected with medium contains puromycin (2.5 μg/mL; Invitrogen), and the stable knock‐down of mindin (shMindin) was confirmed using Western blot analysis.

The mouse mindin overexpression vector was constructed as previously described.^20^ CMT93 and CT26 WT cells which stably overexpressed mindin were selected with G418 (500 μg/mL; Invitrogen). The empty PCMV4 vector was applied as controls (PCMV4). The mRNA and protein expressions of mindin were detected by RT‐PCR and Western blot.

### 2.5 Measurement of mRNA expression

Total RNA was isolated from cells using TRIzol (Invitrogen) according to the protocol. The mRNA expression level was detected using the following primers: mindin forward, 5′‐CAGCCCTGACTGGTTTGTGGGC‐3′ and reverse, 5′‐CCCTGGGACTCTGCTGTAGCCGCACG‐3′; and GAPDH forward, 5′‐TGGCAAAGTGGAGATTGTTGCC‐3′ and reverse, 5′‐AAGATGGTGATGGGCTTCCCG‐3′.

### 2.6 Western blot analysis

We applied the Mammalian Cell Lysis Reagent (Thermo Scientific, Rockford, IL) to extract proteins from cells and tissues according to the protocol. The protein was separated by 10% SDS‐PAGE and then transferred to PVDF membranes. Then blocked with 5% non‐fat milk and incubated with the primary antibodies and followed by the incubation with HRP‐conjugated secondary antibodies. Then detect the protein bands using ECL detection kit (Santa Cruz, CA). Primary antibodies of anti‐ERK1/2 (9102), p‐ERK1/2 (4370), NF‐κB (p65) (8242), p‐NF‐κB (p65) (3033), c‐Fos (4384), FosB (2263), c‐Jun (9165), CDK6 (3136), Cyclin D1 (2922), Cyclin D3 (2936) and P27 (3686) were purchased from Cell Signaling Technology (Boston, MA); anti‐FRA1 (ab124722), CDK4 (ab108357), P15 (ab53034), mindin (ab187920), GAPDH (ab181602) and tubulin (ab210797) from were purchased Abcam (Cambridge, MA), and HRP‐conjugated anti‐rabbit (A8275) and mouse (A5906) secondary antibodies were purchased from Sigma‐Aldrich (St. Louis, MO).

### 2.7 Cell migration assay

The invasive activity of the CMT93‐ and CT26 WT‐based cell lines was analysed using 24‐well polycarbonate filters (BD Biosciences, Bedford, MA). The cell density was adjusted to 1 × 10^6^/mL using serum‐free DMEM medium. The total 200 μL of 1 × 10^6^/mL cell density suspension was added into upper Transwell chamber, and 750 μL of DMEM medium with 10% FBS was added into each lower chamber. Then, the cells were fixed with methyl alcohol after 22‐hour incubation and removed the cells on the upper surface of the filter by wiping with a cotton swab. Then, the invading cells were stained with basic violet and counted under microscope.

### 2.8 CCK‐8 cell proliferation assay

The 2 × 10^3^ cells/well densities of cells were seeded into 96‐well plates. Then, 10 μL of Cell Counting Kit‐8 solution (DoJinDo, Tokyo, Japan) was added into each well after 24, 48, 72 or 96 hours. The absorbance was measured at a wavelength of 450 nm on microplate reader after additional 4‐hour culture.

### 2.9 BrdU cell proliferation assay

The BrdU assays (Cell Signaling Technology) were performed according to the manufacturer's recommended protocol. Briefly, 2 × 10^4^/well density of cells were seeded into 96‐well and incubated for 48 hours. The cells were cultured additional 4 hours after supplement with BrdU into each well. Then, performed the fixing/denaturing, detection antibody, HRP‐conjugated secondary antibody, TMB substrate and STOP Solutions procedures. The absorbance was measured at a wavelength of 450 nm on microplate reader.

### 2.10 Flow cytometry (FCM) analysis

The CT26, CMT93 and RAW264.7 cells were harvested from the plates using PBS buffer (Invitrogen) and stained with an anti‐mouse CD133‐PE antibody (eBioscience, San Diego, CA). The tumour tissue was cut out from the rectum of mice and digested with fluid DNA enzyme I (ROCHE, 101041590001) and collagenase IV (Thermo Fisher, Carlsbad, CA, 17104019) for 1 hour, filter through 70‐μm filter, and additionally lysed with ACK Lysis Buffer (Solarbio, 20190726) for 5 minutes. Stained the dead cells (Thermo Fisher, L34964) and add the sealing solution (anti‐CD16/32, BioLegend, San Diego, CA, 101302), antibodies (anti‐CD11b, BD, 557396; anti‐CD45, BD, 560510; anti‐mouse F4/80, BioLegend, 123110), respectively. Then, the stained cells were analysed using a FACSCalibur flow cytometer (San Jose, CA).

### 2.11 Animals and tumour subcutaneous implantation model

C57BL/6 and BALB/c mice were ordered from the Laboratory Animal Center (Shanghai, China). The mindin‐knockout mice were constructed at animal facility centre of Xiamen University. The mice were housed at SPF environment and were generally used between 6 and 8 weeks of age. CMT cells (5 × 10^6^; CMT93‐shMindin, CMT93‐PU6, CMT93‐Mindin and CMT93‐PCMV4) and CT26 WT cells (5 × 10^6^; CT26 WT‐shMindin, CT26 WT‐PU6, CT26 WT‐Mindin and CT26 WT‐PCMV4) were subcutaneously injected into the C57BL/6 and BALB/c mice, respectively. The ears of each group (n = 5) were tagged for identification. The solid tumours were observed in all subjects after 6 days of cell injections. Each tumour size was monitored once every 3 days for until end point of 24 days using a Vernier calliper. The formula of [length (mm) × width (mm)^2^]/2 was applied to calculate the tumour volume.

### 2.12 Colitis‐associated colorectal cancer (CAC) model

Supernatants containing lentiviral particles for either the overexpression or shRNA‐mediated knock‐down of mindin were ordered from GenePharma (Shanghai, China). The ≤1 × 10^9^ (TU)/mL titres of lentiviral stock were according to the previous report.^32^, ^33^ A volume of 200 μL of lentiviral supernatant was injected into the veins of C57BL/6 mice tail and repeated once in the fifth week. One week after the administration of the viral supernatants, the study mice were i.p. injected with concentration of 12.5 mg/kg AOM in saline. One week after the AOM injection, administered mice with 3% DSS in drinking water for 5 days, and switched to water for 16 days. Then, repeated this procedure for three cycles and harvested the colon tissues at day 77.

### 2.13 Immunohistochemistry

The rectum tissue of the mice was fixed with formalin and embedded with paraffin. We treated the sections with peroxidase, followed by blockage of donkey serum. The sections were incubated with an anti‐mindin antibody (Santa Cruz, USA) overnight at 4°C. Then supplied the secondary antibodies and detected by DAB Kit (Maxim, Fuzhou, China). The haematoxylin was used in counterstaining.

### 2.14 Enzyme‐linked immunosorbent assay

ELISA assays of the mindin (Cloud‐Clone Corp, Katy, TX) were performed on serum samples and measured the absorbance using a microplate reader with a wavelength of 450 nm.

### 2.15 Statistic analysis

The SPSS 17.0 software (SPSS Inc, Chicago, IL) was applied at statistic analysis, and the comparison among study groups used Student's *t* test. All values are expressed as SD, and *P* < 0.05 was considered to be statistically significant.

## 3 RESULTS

### 3.1 Mindin attenuates colon cancer cell proliferation in vitro

**FIGURE 1:**
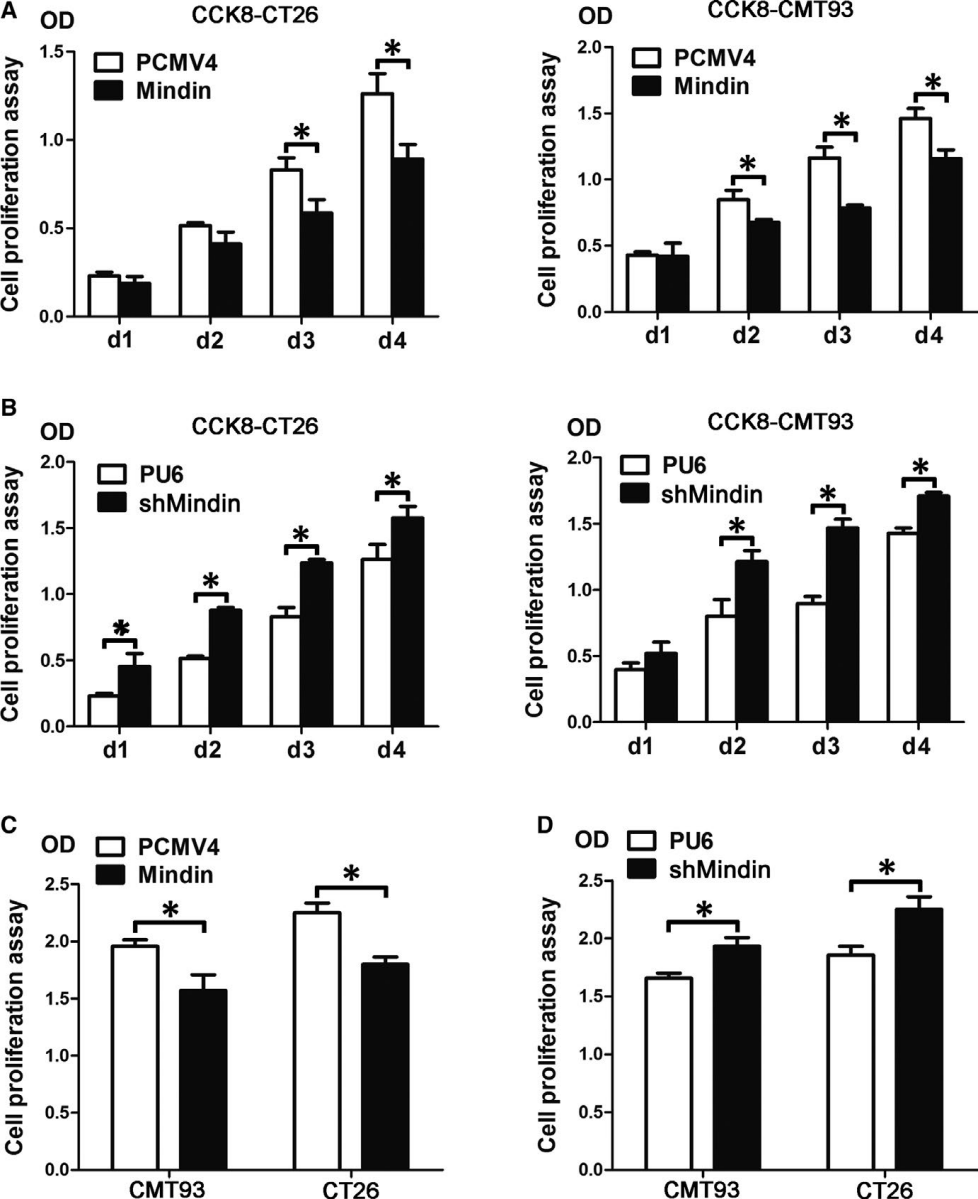
Mindin suppresses colon cancer cell proliferation in vitro. A, Analysis of CT26 WT (left side) and CMT93 (right side) cell proliferation in the mindin‐overexpressing cells and control cells by CCK‐8 assay (*P < 0.05). B, Analysis of cell proliferation in the mindin knock‐down cells and control cells by CCK‐8 assay (*P < 0.05). C, Analysis of cell proliferation in the mindin‐overexpressing cells and control cells by BrdU assay (*P < 0.05). D, Analysis of cell proliferation in the mindin knock‐down cells and control cells by BrdU assay (*P < 0.05)

To determine the function of mindin in colon cancer cells, we used the mouse syngeneic colorectal cancer cell lines CMT93 and CT26 WT to establish stable cell pools with either mindin overexpression or knock‐down as well as controls transfected with empty vector. As shown in Figure S1A‐D, mindin was successfully overexpressed or knocked‐down in the CMT93 and CT26 WT cell lines, as assessed at both the protein and mRNA levels. Next, we performed the CCK‐8 (Figure 1A,B) and BrdU (Figure 1C,D) proliferation assays and FCM analysis of CD133 expression (Figure S2A‐D) on the cell lines to determine the role of mindin in colorectal cancer cell proliferation. Our data from all three assays consistently demonstrated that the overexpression of mindin suppressed the proliferation of both the CMT93 and CT26 WT colon cancer cells in comparison with the controls (*P* < 0.05). In contrast, the silencing of mindin promoted cancer cell proliferation (*P* < 0.05) in vitro*.* In addition, we performed cell migration assays and found that the invasive ability of the cells was significantly decreased with the overexpression of mindin and increased with the silencing of mindin compared with the controls (Figure S3A‐D, *P* < 0.05). These results reveal the novel tumour suppressive function of mindin during colon cancer cell proliferation in vitro.

### 3.2 Mindin suppresses in vivo tumour growth in a subcutaneous implantation tumour model

**FIGURE 2:**
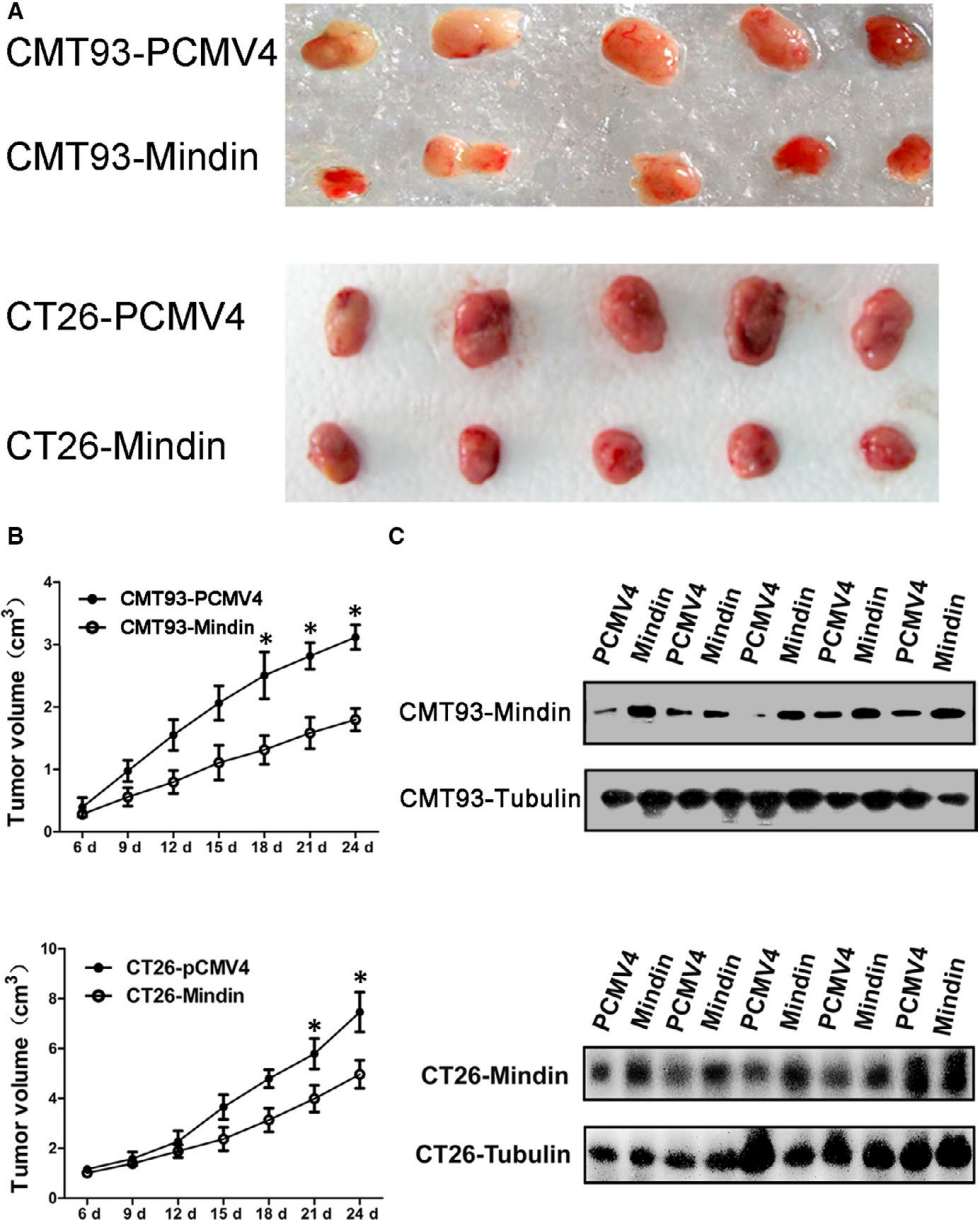
Subcutaneous implantation tumour growth of mindin‐overexpressing CMT93 and CT26 WT cells. C57BL/6 and BALB/c mice were subcutaneously injected with stable mindin‐overexpressing CMT93 or CT26 WT cells, or PCMV4 control cells. Tumour size was measured every 3 d for 24 d. A, Images of isolated tumours from the four groups of study mice (n = 5). B, In vivo tumour growth resulting from the mindin‐overexpressing CMT93 or CT26 WT cell (n = 5, *P < 0.05). C, Western blot analysis confirming mindin protein overexpression in the tumour tissues of the four study groups at the end of the study period. Tubulin was used as a protein loading control (n = 5). Upper panel indicates CMT93, and lower panel indicates CT26 WT among A‐C

**FIGURE 3:**
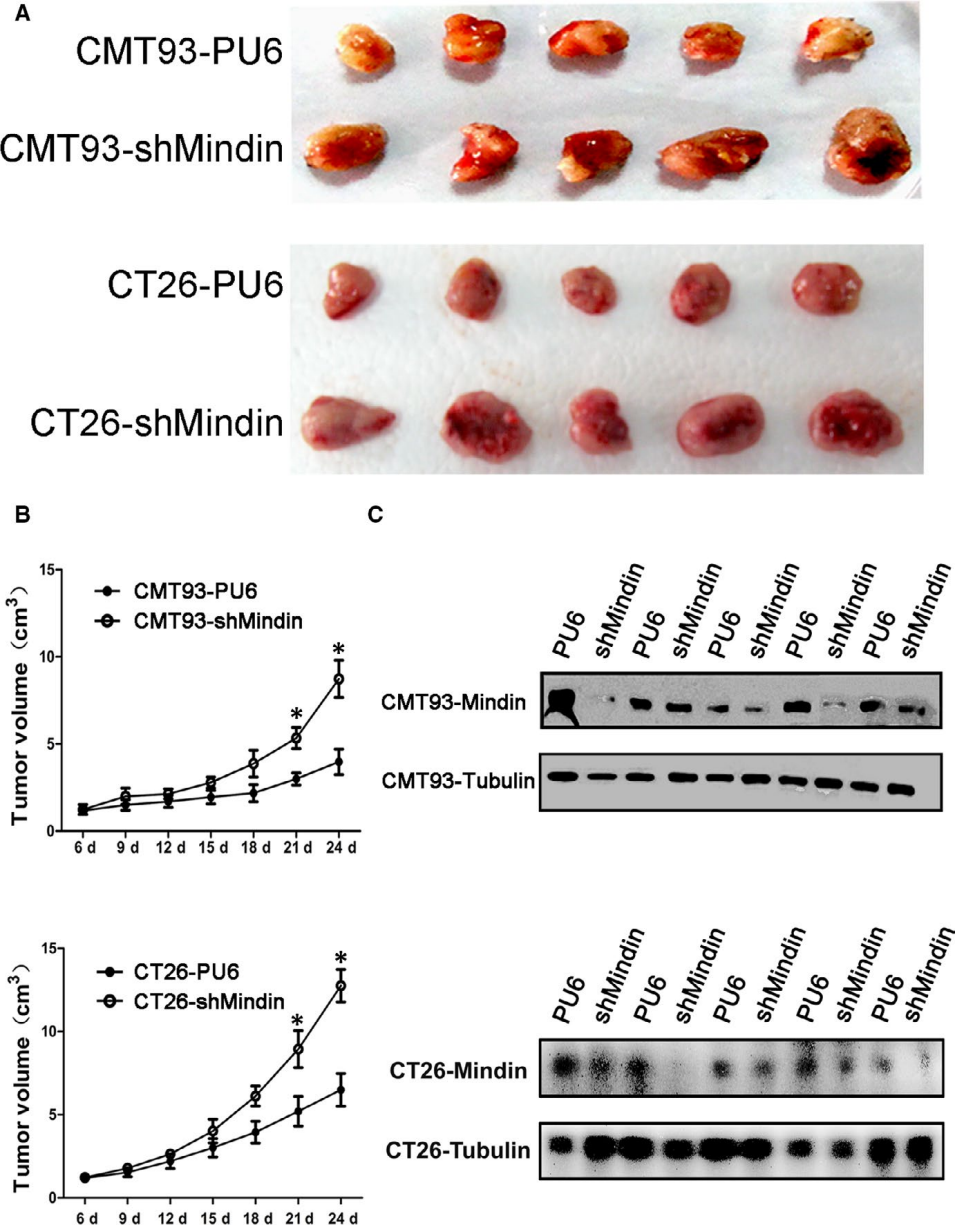
Subcutaneous implantation tumour growth of mindin knock‐down CMT93 and CT26 WT cells. C57BL/6 and BALB/c mice were subcutaneously injected with stable mindin knock‐down CMT93 or CT26 WT cells, or PU6 control cells. Tumour size was measured every 3 d for 24 d. A, Images of isolated tumours from the four groups of study mice (n = 5). B, In vivo tumour growth resulting from the mindin knock‐down CMT93 or CT26 WT cell (n = 5, *P < 0.05). C, Western blot analysis confirming mindin protein deficiency in the tumour tissues of the four study groups at the end of the study period. Tubulin was used as a protein loading control (n = 5). Upper panel indicates CMT93, and lower panel indicates CT26 WT among A‐C

To determine the role of mindin during colon cancer progression in vivo, we subcutaneously injected CMT93 and CT26 WT cells with or without mindin overexpression or knock‐down into C57BL/6 and BALB/c mice. Interestingly, the overexpression of mindin significantly suppressed the growth of the CMT93 and CT26 WT subcutaneous tumours when compared with the control groups (Figure 2A,B). Moreover, the knock‐down of mindin significantly promoted tumour growth in the mice with CMT93 and CT26 WT cell (Figure 3A,B). To confirm the maintenance of the overexpression and knock‐down of the mindin protein, we killed the study mice at day 24, extracted the protein from the tumour tissues and analysed mindin expression using Western blot analysis. As shown in Figures 2C and 3C, the overexpression and knock‐down of the mindin protein was maintained until the end of the study period.

### 3.3 Mindin plays a suppressive role in a colitis‐associated colon cancer model

**FIGURE 4:**
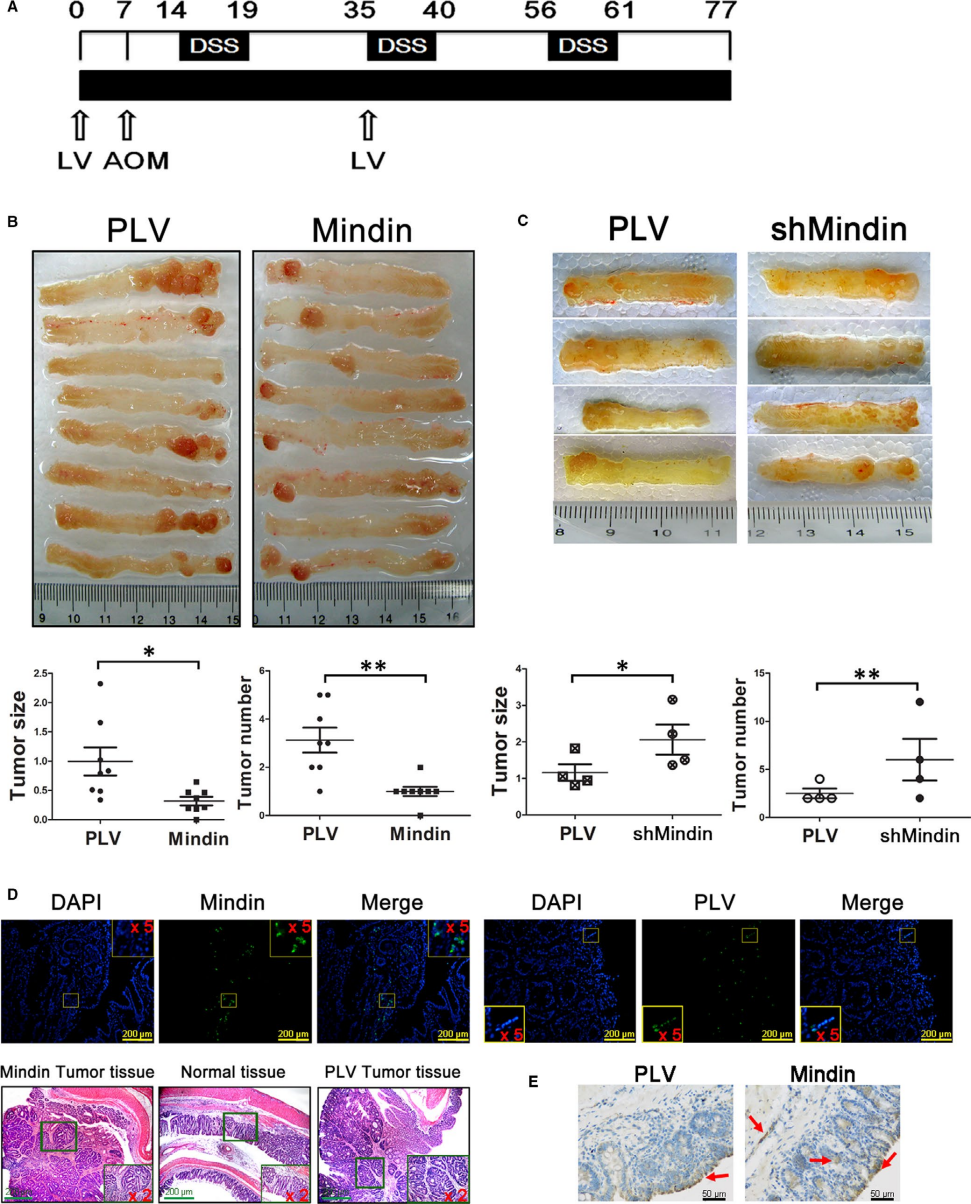
Lentivirus‐mediated colitis‐associated cancer model. A, Experimental protocol used to induce CAC and the administration of lentiviral vectors. B, Images of isolated colon tissue from the mindin‐overexpression groups and control mice at the end of the study period (upper panel, n = 8), and tumour size and number (lower panel, n = 8, *P < 0.05, **P < 0.01). C, Photograph of isolated colon tissue from the mindin knock‐down groups and control mice (upper panel, n = 4), and tumour size and number (lower panel, n = 4, *P < 0.05, **P < 0.01). D and E, Confocal microscopy and anti‐mindin immunohistochemistry analysis of frozen and paraffin‐embedded colon sections showing the GFP reporter for lentiviral vector expression and mindin protein (as shown by the red arrows), and H&E staining of serial sections of mouse CAC tissues

Mindin has been studied as important factor in the immune response, and the connection between inflammation and tumorigenesis is well established. To further determine the function of mindin on CAC development, we administered lentiviral vector‐mediated overexpression or knock‐down of mindin in an AOM/DSS‐induced CAC model (Figure 4A). On day 77, a significant decrease in tumour size and number was observed in the mindin‐overexpressing mice compared with the control group (Figure 4B, *P* < 0.05). At the end point of the study, the efficiency of lentivirus transduction was indicated by GFP reporter and anti‐mindin immunohistochemistry analysis and the characteristics of the tumours were confirmed using HE staining (Figure 4D,E). We utilized the same system and observed significant increases in tumour size and number in the mindin knocked‐down mice (Figure 4C, *P* < 0.05).

### 3.4 Mindin regulates colon cancer cell proliferation via the p‐ERK and c‐Fos signalling pathways and cell cycle control

**FIGURE 5:**
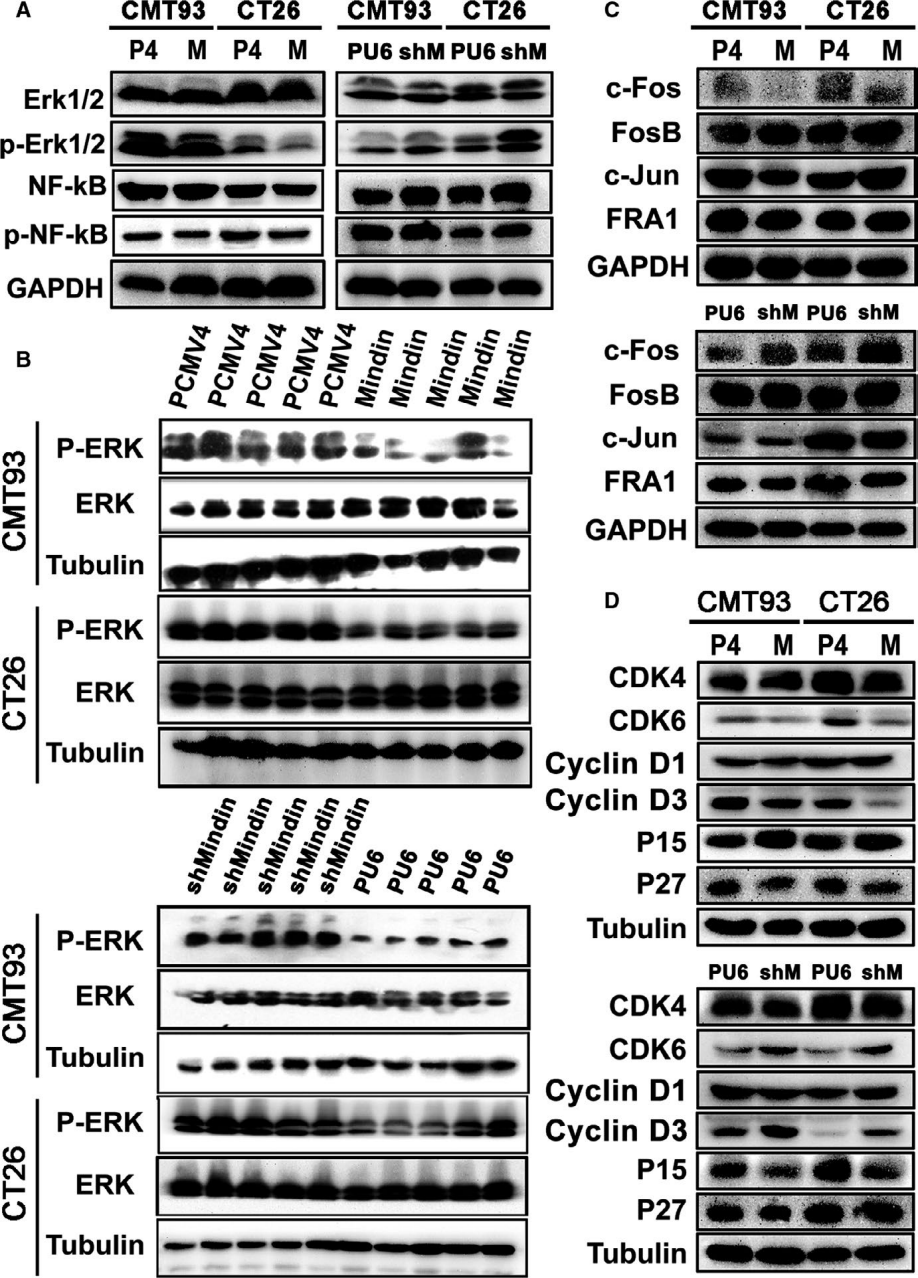
Mindin‐mediated signalling pathway analysis*.* A, Western blot analysis using antibodies against ERK1/2, phospho‐ERK1/2, p65‐NF‐κB and phospho‐p65‐NF‐κB and protein lysates from mindin, PCMV4, shMindin and PU6 cells. GAPDH was used as a loading control. B, Western blot analysis of the phosphorylation level of ERK1/2 in mindin‐overexpressing (upper panel) or knock‐down (lower panel) tumour tissues from tumour subcutaneous implantation model mice. Tubulin was used as a protein loading control (n = 5). C and D, Western blot analysis of c‐Fos, FosB, c‐Jun, FRA1, CDK4, CDK6, CyclinD1, CyclinD3, P15 and P27 expression in the stable cell lines and their controls

To define the signalling pathways involved in the mindin‐mediated regulation of colorectal cancer cell proliferation, we analysed the protein expression of ERK/p‐ERK and NF‐κB/p‐NF‐κB in the mindin overexpression or knock‐down CMT93 and CT26 WT cell lines and the control cell lines. Our data showed that the phosphorylation level of ERK1/2 was down‐regulated in the mindin‐overexpressing cells and up‐regulated in the mindin knock‐down cells (Figure 5A). No significant differences in NF‐κB phosphorylation were observed in these cells (Figure 5A). We further analysed the phosphorylation of ERK1/2 in the tumour tissues that were induced from the mindin‐overexpressing and knock‐down cells in the in vivo transplantation model. Consistent with the in vitro study, the phosphorylation level of ERK1/2 was down‐regulated in the mindin‐overexpressing tumour tissues and up‐regulated in the mindin knock‐down tumour tissues at the end of the study period (Figure 5B).

**FIGURE 6:**
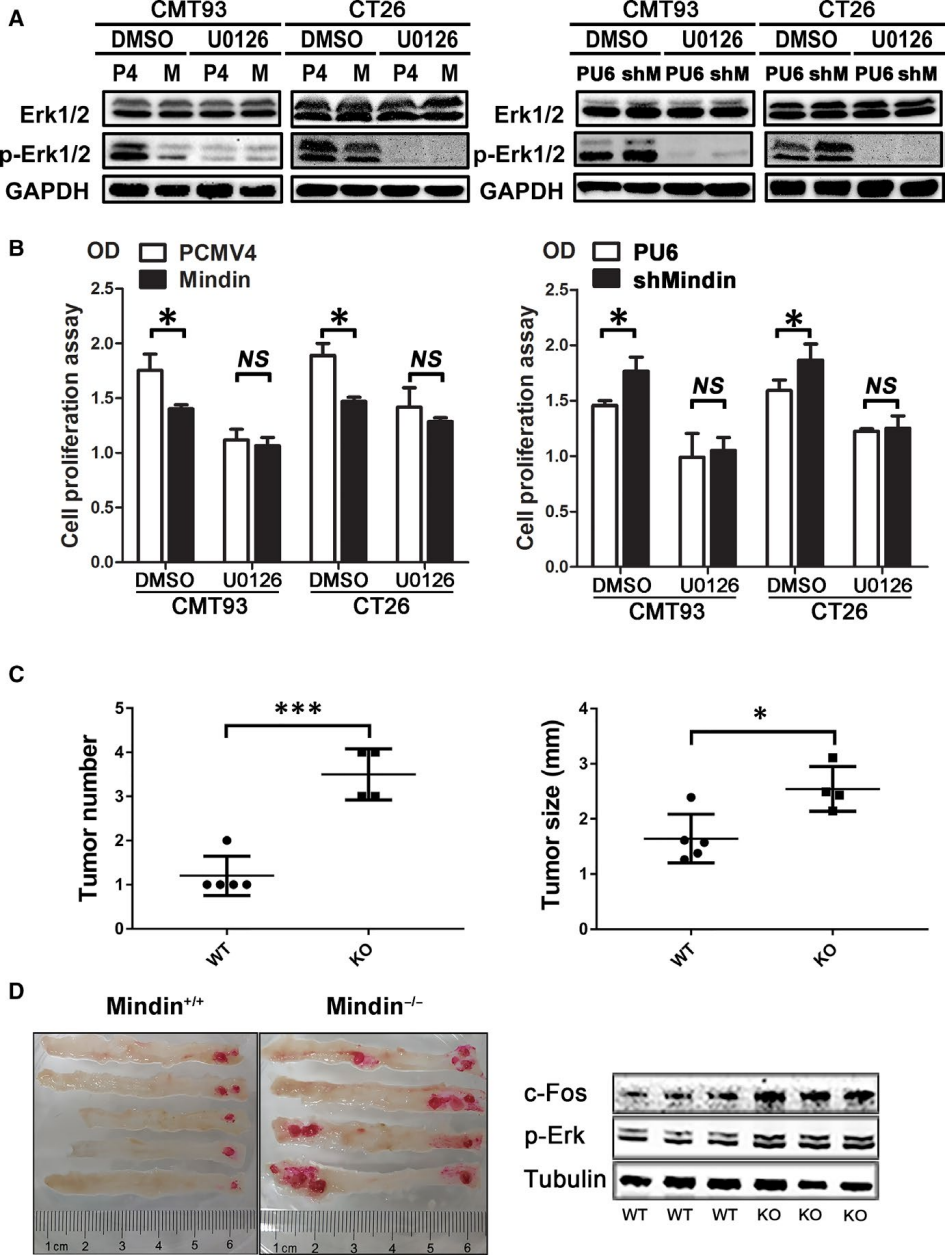
U0126 inhibition of ERK1/2 phosphorylation, cell proliferation and colitis‐associated cancer model of mindin‐knockout mice. A, Western blot analysis of U0126‐treated cells using antibodies against ERK1/2 and phospho‐ERK1/2. GAPDH was used as a loading control. B, Analysis of U0126‐treated cell proliferation in the mindin‐overexpressing (left panel) or knock‐down (right panel) and control cells by BrdU assay (*P < 0.05). C, Tumour number (left panel) and size (right panel) of isolated colon tissue from the mindin‐knockout groups and control mice at the end of the study (n = 8, *P < 0.05, ***P < 0.01). D, Representative images of isolated colon tissue from the mindin‐knockout groups and control mice at the end of the study (left panel). Western blot analysis of the phosphorylation level of ERK in mindin‐knockout or control tumour tissues from CRC model mice (right panel). Tubulin was used as a loading control

To examine whether inhibition of ERK1/2 phosphorylation affects colon cancer cell proliferation, we cultured mindin overexpression or knock‐down CMT93 and CT26 WT cell lines and the control cells in the presence of U0126, a specific inhibitor of MEK pathway. We observed that U0126 significantly inhibited ERK1/2 phosphorylation (Figure 6A) and cell proliferation (Figure 6B) compared with the control. In addition, the cell proliferation of mindin‐overexpression group has no significant difference with the control group after cells treated with U0126. However, there was a significant decrease in cell proliferation in mindin‐overexpressing CMT93 and CT26 cells treated with DMSO compared with the control cells (Figure 6B, left panel). Taken together, mindin regulates cancer cell proliferation in vitro and in vivo via a MAPK/ERK‐mediated signalling pathway.

Next, we analysed the expression of the Fos proteins, which are induced by a variety of extracellular stimuli. As shown in Figure 5C, c‐Fos protein expression was decreased in the mindin‐overexpressing cells and increased in the mindin knock‐down cells. However, we did not observe significant differences in the expression of the FosB, FRA1 or c‐Jun proteins between these cells and the controls.

Furthermore, we analysed the expression of various cell cycle regulation proteins that are directly associated with the progression of cell proliferation. Our results indicated that the protein levels of CyclinD3 and CDK6 were down‐regulated in the mindin‐overexpressing cells and up‐regulated in the mindin‐deficient cells (Figure 5D; Figure S5A). Moreover, the expression of the P15 protein was increased in the mindin‐overexpressing cells and decreased in the mindin‐deficient cells (Figure 5D). No significant differences in the expression of CyclinD1 or CDK4 were observed in these cells.

### 3.5 Mindin deficiency promotes tumour growth in vivo

**FIGURE 7:**
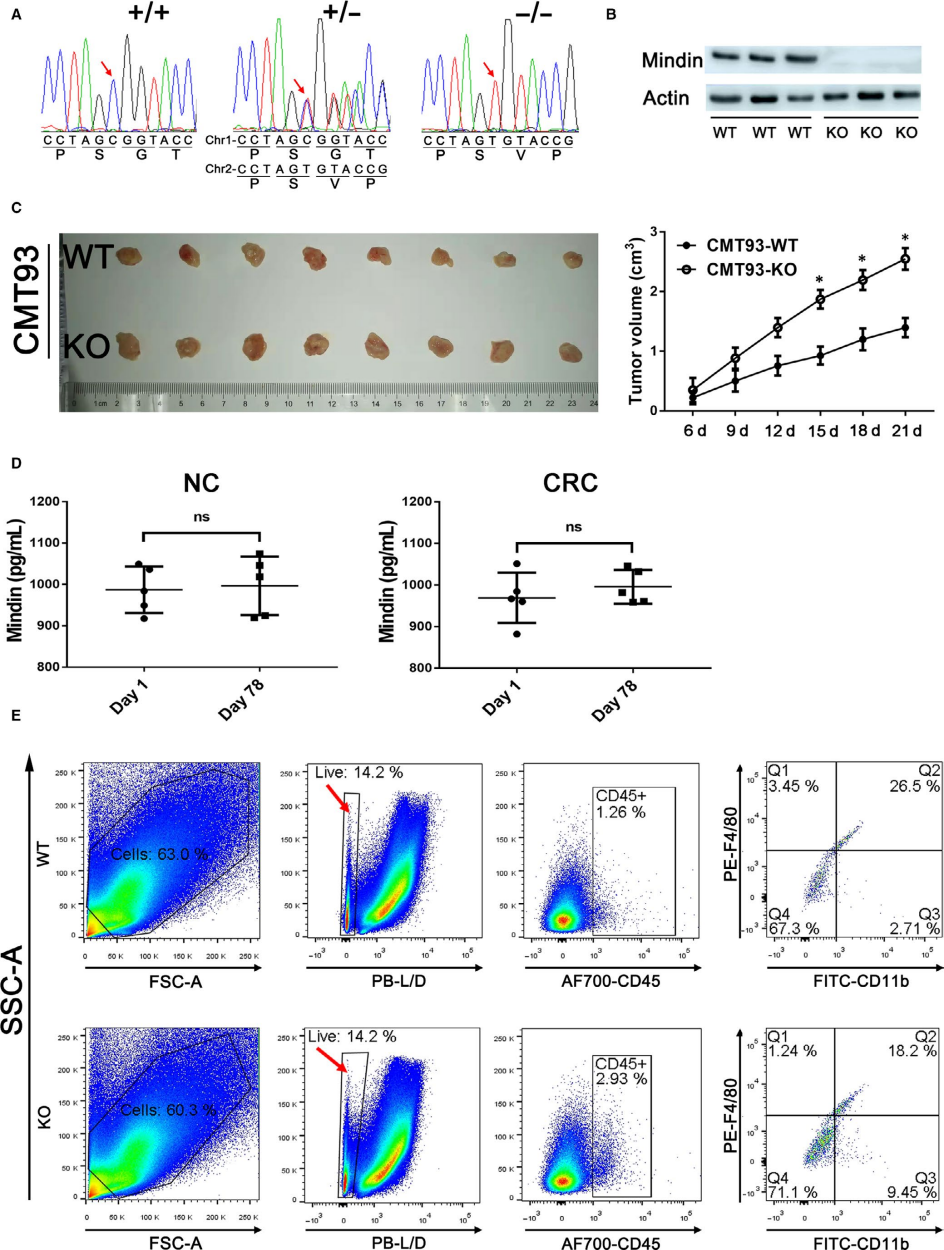
A, Sequencing chromatograms show the nucleotide mutation of mindin−/− mice using a CRISPR‐Cas system. B, Western blot analysis using antibody against mindin on mice colon tissues. Actin was used as a loading control. C, The tumour images of 21 d after subcutaneous injection of CMT93 colorectal cancer cells in mindin‐knockout and the control mice. Tumour size was measured and quantitatively analysed (n = 5, *P < 0.05). D, Mindin expression was measured by ELISA in the first day and the end point of the model in WT mice serum with or without CRC procedure. E, The cells were isolated from tumour tissues of the AOM/DSS‐induced CRC mice. The procedure of flow cytometry analysis as follows: gated the single cells first, separated cells with the LIVE/DEAD dye and gated the CD45^+^ cells, then right panels showed the FITC‐CD11b and PE‐F4/80‐stained cells

To examine whether the mindin gene had been knocked‐out, we performed the sequencing chromatograms and the Western blot analysis and confirmed the mindin deficiency in Figure 7A,B. As shown in Figure S5C, the bases of CG in WT mice were replaced to be T base in mindin‐KO mice (as shown by the red arrow) and resulted in a shiftment of earlier stop codon (as shown by the black arrow). To further determine the function of mindin on CAC development, we conducted the AOM/DSS‐induced CRC model in the mindin‐knockout and the control mice. On day 77, a prominent increase in tumour size and number was observed in the mindin‐knockout mice compared with the control group (Figure 6C and left penal of 6D, *P* < 0.05). Consistent with the above results, the protein level of c‐Fos and p‐Erk protein was up‐regulated in the mindin KO tumour tissues and down‐regulated in the WT tumour tissues (right penal of Figure 6D). We further analysed the expression of mindin on tumour and adjacent normal colon tissues from CAC model of WT mice. However, we did not observe the significant difference in mindin protein expression (Figure S4C). To observe tumour formation in mindin‐knockout and control mice, we grafted CMT93 colorectal cancer cells in mindin‐knockout and control mice. As shown in Figure 7C, the significant larger tumour volumes were observed in mindin‐knockout mice compared with the control group. This is consistent with our results from colitis‐associated colon cancer model. To investigate the changes in mindin expression in mice serum with or without CAC procedure, we performed the ELISA assays. As shown in Figure 7D, there were no significant changes in secreting mindin in WT mice serum with or without CAC procedure.

## 4 DISCUSSION

The microenvironment of colorectal tumours has gained increasing attention because it is involved in tumour initiation, progression and metastasis.^34^ Components of the ECM are known to regulate critical cellular processes, such as growth, differentiation, apoptosis and carcinogenesis.^35^, ^36^, ^37^ The ECM protein mindin has been shown to be expressed in primary lung cancers, and its homolog in humans, Spondin 2, is a prostate‐associated protein.^38^, ^39^ Mindin inhibits vascular hyperplasia by suppression of abnormal vascular smooth muscle cell proliferation, migration and phenotypic switching.^40^ Furthermore, mindin has been used as a potential therapeutic target for hypertrophy and heart failure.^41^, ^42^ In this study, the overexpression of mindin suppressed colon cancer cell proliferation and the silencing of mindin promoted cancer cell proliferation in vitro*.* We also explored how mindin functions in vivo. Our data showed that the overexpression of mindin significantly suppressed tumour growth in an in vivo transplantation model, and this regulatory process was consistent in an AOM/DSS‐induced CAC model that was subjected to lentiviral vector‐mediated mindin overexpression. Furthermore, the silencing of mindin using knockout and knock‐down methods reversed this phenotype in both murine colon cancer models. Mindin was reported as a tumour‐promoting factor by Schmid et al on employment of human cell lines in vivo.^24^ However, we previously determined that mindin attenuates CRC progression by blocking angiogenesis through Egr‐1–mediated regulation, and did not observe the direct suppression of human cancer cell proliferation and colony formation ability.^26^ Indeed, we used the mice syngenic cell lines and mindin‐deficient mice in this study. Our data were contrary to previous report that mindin up‐regulation was shown to be poor survival indicator of colorectal cancer patients.^24^, ^43^ As well known, different regions, races and lifestyles may cause differences in the development and progression of colon cancer.^44^, ^45^, ^46^ In our previous study, the colorectal cancer patients are mainly from Fujian province, south‐east coastal area of China. The CRC tumour suppressive phenotype of mindin in this mice study was consistent with our previous human mindin study.^26^ Nevertheless, tumour suppressive function of mindin is mechanically differed in mice and human study. The mindin amino acid sequences from Homo sapiens and Mus musculus contain 90.1% in similarity and 84.0% in identity. The difference in amino acid sequences may induce the structural changes resulted in the different mechanisms of mindin attenuating the CRC progression.

The mechanism responsible for the attenuation of colorectal cancer cell progression by mindin is partially mediated by the phosphorylation of ERK. Mindin serves as both an integrin ligand and a pattern recognition molecule.^14^, ^47^ We recently reported that mindin acts as a ligand of integrins CD11b/CD18.^19^ However, our flow cytometry analysis has shown that both of CMT93 and CT26 colon cancer cell lines did not express the CD11b and CD18 integrins compared with the positive control of RAW264.7 macrophage cell line (Figure S4A). According to the “cancer immunoediting” hypothesis, the immune system played a dual role of host‐protecting and tumour‐sculpting in during tumour progression.^48^ In the escape phase, alterations leading to reduced immune recognition at the tumour cell level such as a loss of antigens promote tumour outgrowth.^49^ Thus, the proliferation of CMT93 and CT26 cancer cells might be integrin‐independent manner. To investigate whether the involvement of integrin‐mediated function, we isolated the cells from tumour tissues of the AOM/DSS‐induced CRC mice and performed the flow cytometry analysis. As shown in Figure 7E, the tumour surrounding F4/80^+^ macrophages expressed integrin CD11b, and the percentage of macrophages that express CD11b from WT mice is significantly higher than mindin‐KO mice. This result suggests the requirements of further study in the future, such as study on mindin and integrin double deficiency mice with rescue experiments.

ECM proteins contribute to proliferation, adhesion and migration by functioning as integrin ligands.^50^ Integrins coordinate with growth factors for the phosphorylation of receptor tyrosine kinases, MAPK/ERK activation and the regulation of cell proliferation.^51^, ^52^ Our data showed that the phosphorylation level of ERK1/2 was down‐regulated in mindin‐overexpressing cells and up‐regulated in mindin‐deficient cells. NF‐kB is involved in the pathogenesis of colorectal cancer^53^; however, no significant difference in p‐NF‐kB was observed in our experiments. Additionally, the activation of Fos proteins by ERK kinases in response to extracellular stimuli may further increase transcriptional activity.^54^, ^55^ We found that c‐Fos protein expression was inhibited by the overexpression of mindin, which induced the down‐regulation of p‐ERK1/2. To examine whether correlation between mindin expression and MAPK activation is observed in human colorectal tumour, we performed the Western blot analysis on the colorectal tumour and paired normal samples from patients. As shown in Figure S4B, our data did not show the significant correlation between mindin expression and MAPK activation. Meanwhile, our previous study showed that mindin attenuates colon cancer progression by blocking angiogenesis via Egr‐1–mediated regulation in human cancer cell lines.^26^

Furthermore, our data indicated that the protein levels of CyclinD3 and CDK6, which regulate the restriction point of mid‐late G1 phase in the cell cycle, were down‐regulated and that the expression of the P15 protein, which selectively inhibits CDK4/6 activity, was increased in mindin‐overexpressing cells.

Mindin was reported as a potential marker for the early detection of ovarian and prostate cancers.^56^, ^57^ In our cancer patient analysis, our data demonstrated that the serum levels of mindin were significantly decreased in colorectal cancer patients compared with healthy controls. Moreover, decreased serum levels of mindin were significantly associated with the early stages of disease.^26^ Interestingly, P Chandrasinghe1 et al identified the mindin/MACC1 axis as a potential therapeutic target for CAC.^58^

Among the immune cells recruited to the tumour site, macrophages are particularly abundant and present at all stages of tumour progression, and most tumour‐associated macrophages (TAMs) promote many properties of tumour progression, including tumour growth, metastasis and angiogenesis.^59^ As shown in Figure 7E, the tumour surrounding F4/80+ macrophages expressed integrin CD11b, and the percentage of macrophages that express CD11b from WT mice is significantly higher than mindin‐KO mice. Thus, mindin might be involved in a new mechanism for the function of macrophages on tumour progression.

This investigation demonstrates the direct tumour suppressive function of mindin during colon cancer development through the regulation of the p‐ERK and c‐Fos signalling pathways and cell cycle control in mice, suggesting that mindin can be used as a therapeutic target for CRC.

## CONFLICT OF INTEREST

The authors declare no conflict of interest.

## AUTHOR CONTRIBUTIONS

GB and RJL designed the experiments; CXS, FYY, XCX, HYN, OXM, LLY, LY and WJF performed the experiments; and GB wrote the paper.

## Supporting information

Fig S1

Fig S2

Fig S3

Fig S4

Fig S5

## Data Availability

I confirm that my article contains a Data Availability Statement even if no data are available unless my article type does not require one.
