# Supplementary figures and images for: Mindin serves as a tumour suppressor gene during colon cancer progression through MAPK/ERK signalling pathway in mice
Source: J Cell Mol Med. 2020 Jul 2;24(15):8391–404. doi: 10.1111/jcmm.15332 (PMC7412704; doi:10.1111/jcmm.15332)

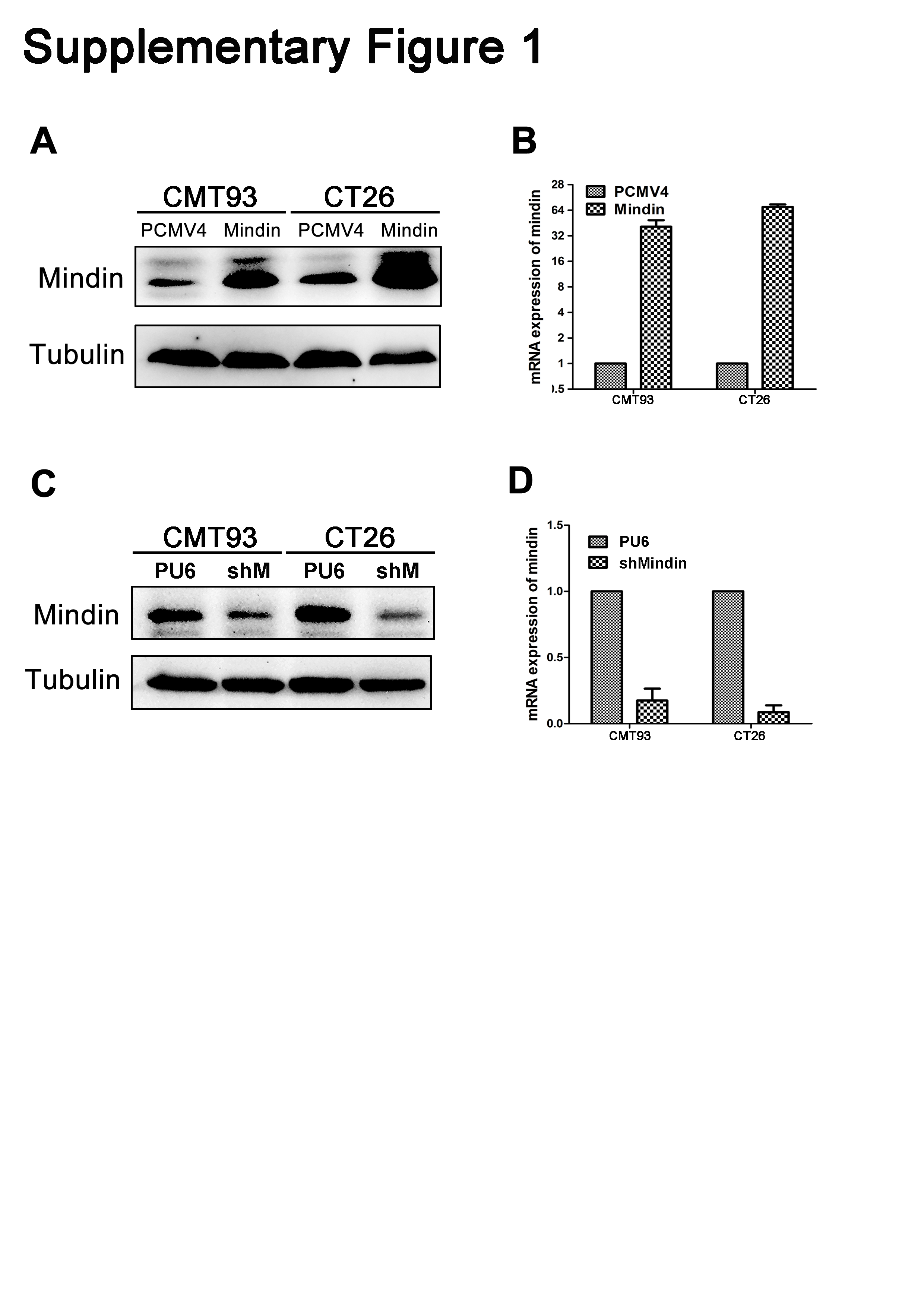

Supplement: Supplementary file 1 — Fig S1 [file JCMM-24-8391-s001.tif]

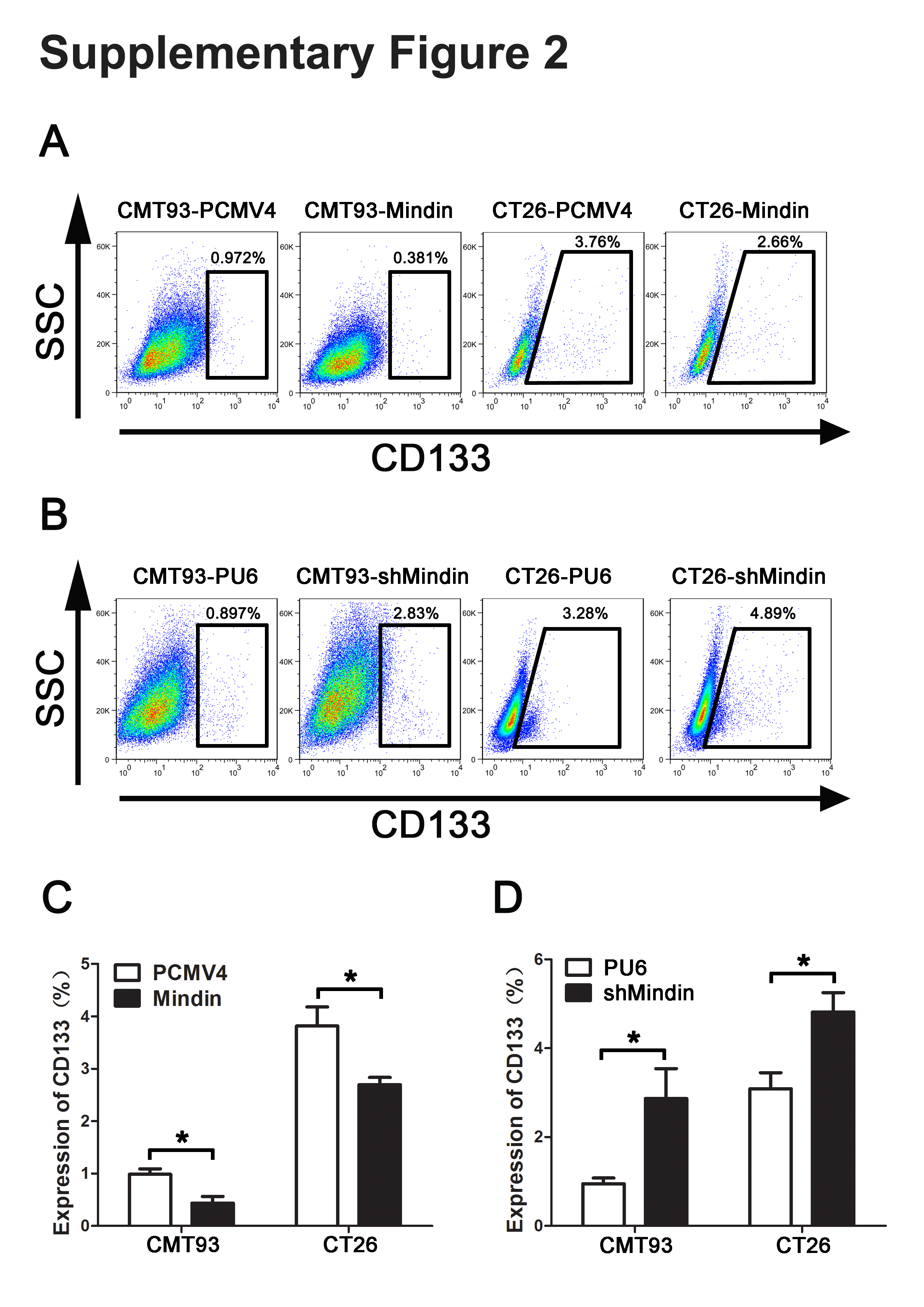

Supplement: Supplementary file 2 — Fig S2 [file JCMM-24-8391-s002.tif]

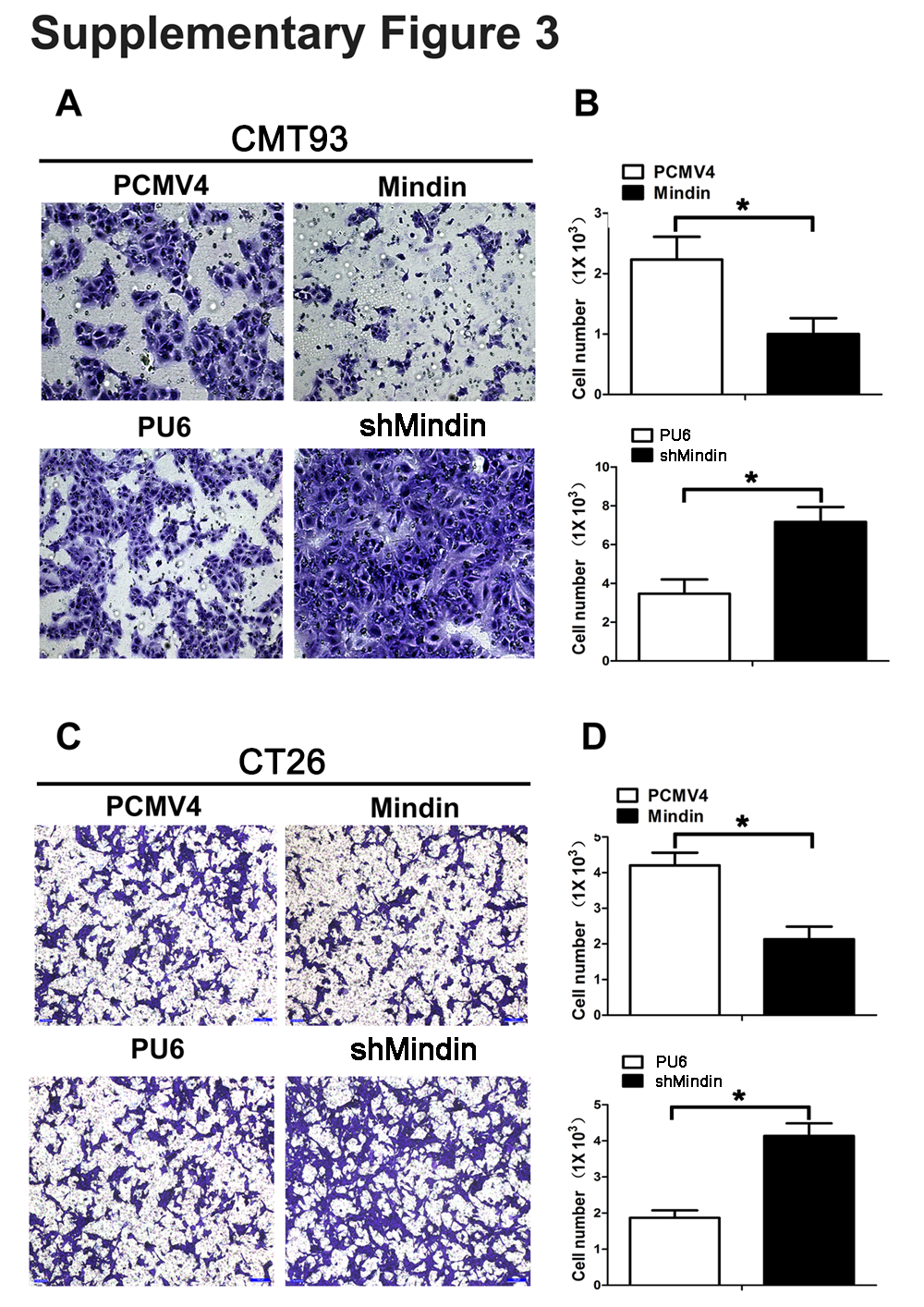

Supplement: Supplementary file 3 — Fig S3 [file JCMM-24-8391-s003.tif]

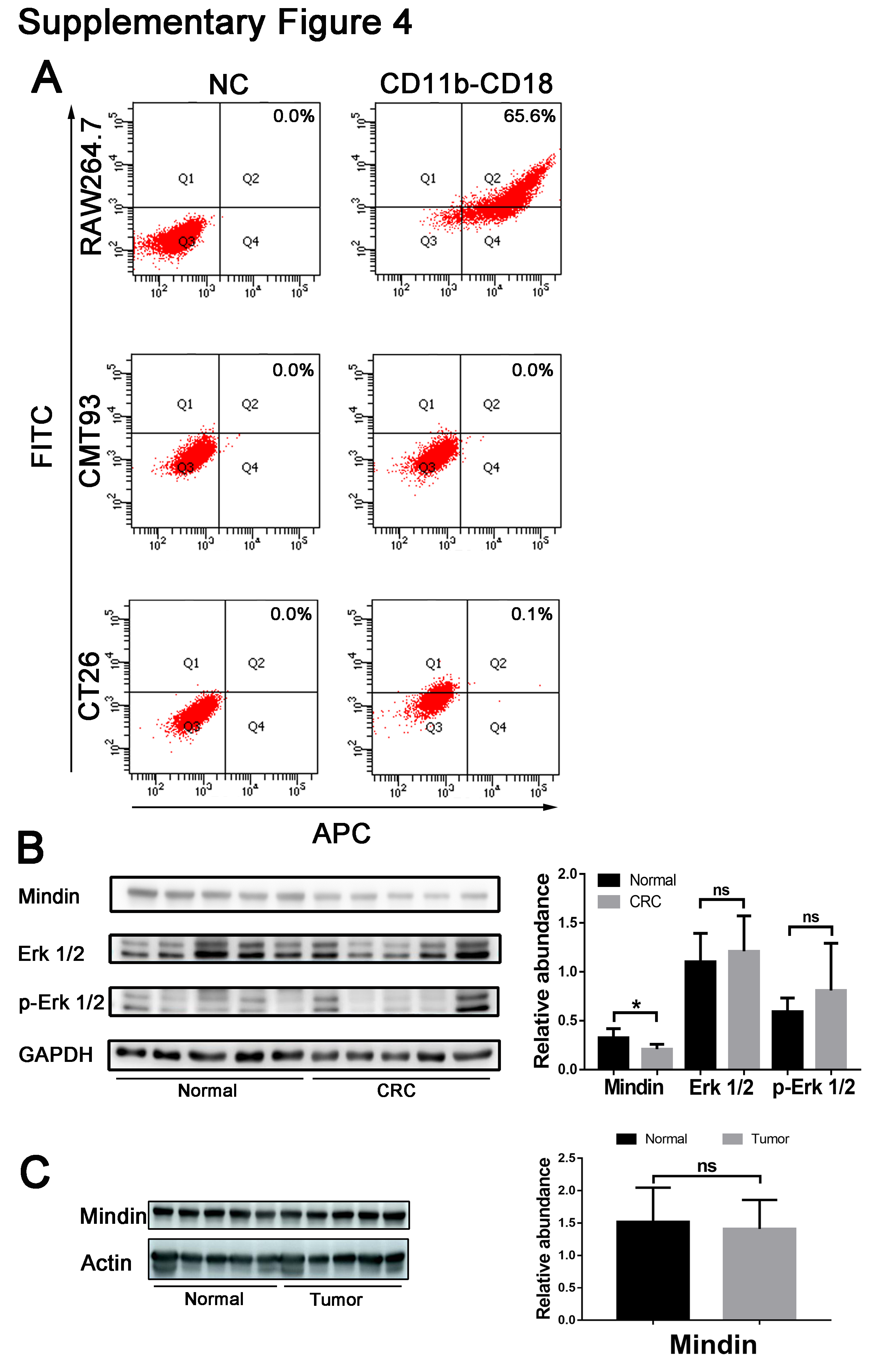

Supplement: Supplementary file 4 — Fig S4 [file JCMM-24-8391-s004.tif]

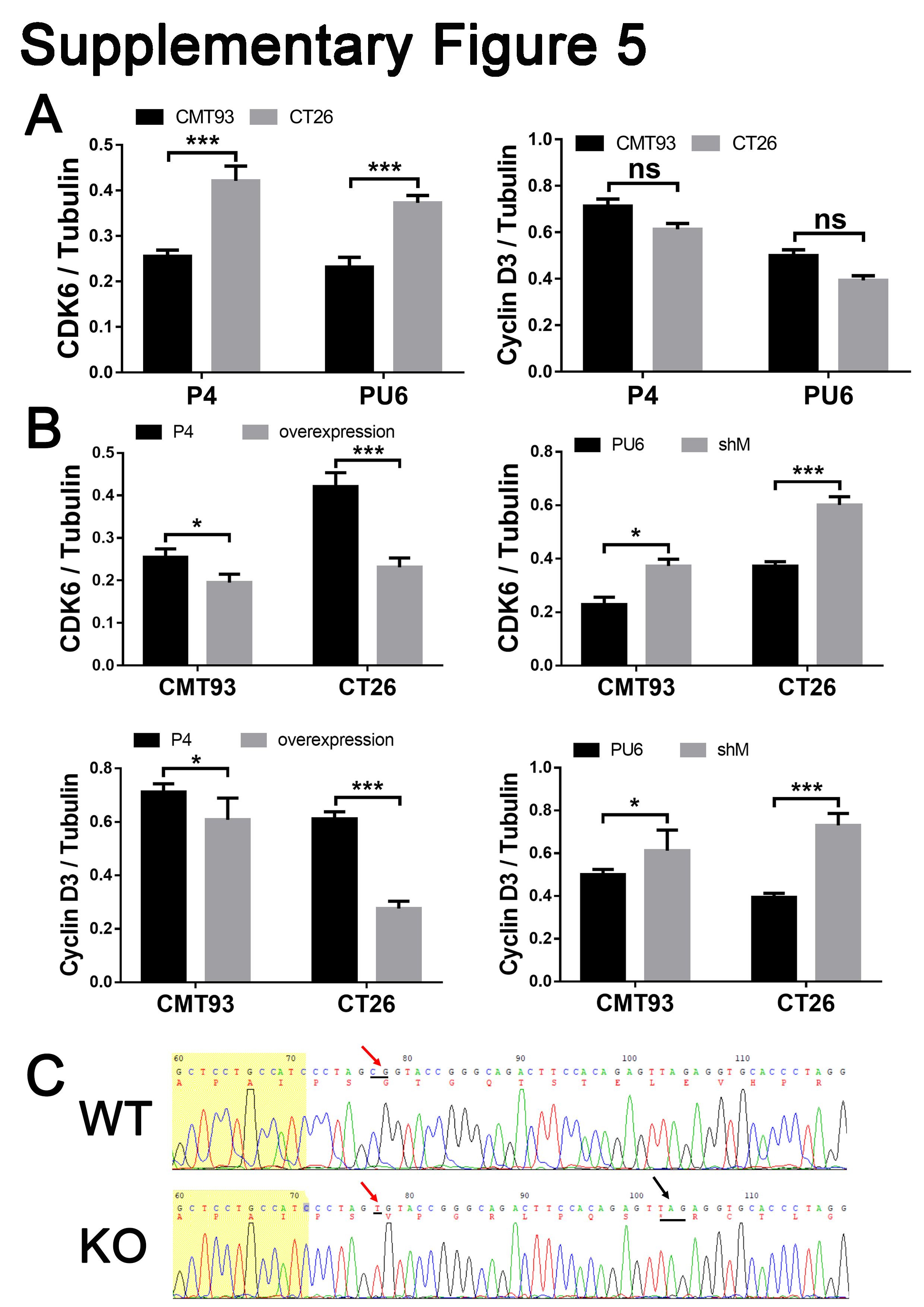

Supplement: Supplementary file 5 — Fig S5 [file JCMM-24-8391-s005.tif]
