# Supplementary material for: Mindin serves as a tumour suppressor gene during colon cancer progression through MAPK/ERK signalling pathway in mice
Source: J Cell Mol Med. 2020 Jul 2;24(15):8391–404. doi: 10.1111/jcmm.15332 (PMC7412704; doi:10.1111/jcmm.15332)
Supplement: Supplementary file 6 [file JCMM-24-8391-s006.docx]

**Fig. S1** Establishment of the mouse syngeneic colorectal cancer cells with stable Mindin knock-down or overexpression. (A) Western blot and (B) RT-PCR analysis of the stable overexpression of Mindin in two cell lines (CMT-Mindin; CT26 WT-Mindin) and their respective controls (CMT-PCMV4; CT26 WT-PCMV4). (C) Western blot and (D) RT-PCR analysis of the stable silencing of Mindin in two cell lines (CMT-siMindin; CT26 WT-siMindin) and their respective controls (CMT-PU6; CT26 WT-PU6). Tubulin was used as a protein loading control.

**Fig. S2** FCM analysis of CD133 expression *in vitro*. (A and C) CD133 expression in the Mindin-overexpressing and control cells (**P*<0.05). (B and D) CD133 expression in the Mindin knock-down and control cells (**P*<0.05).

**Fig. S3** Cell migration assay. Invasion activities of the Mindin-overexpressing and PCMV4 control cells and the Mindin knock-down and PU6 control cells. The CMT cells are shown in A and B, and the CT26 WT cells are shown in C and D. The experiments were performed in triplicate wells. The results are quantitatively presented as histograms on the right, and representative images are shown on the left (**P*<0.05).

**Fig. S4** (A) Flow cytometry analysis of RAW264.7, CMT93 and CT26 cells with anti-CD11b and anti-CD18 antibodies. (B) Western blot analysis (left panel) and quantitative densitometric analysis (right panel) of mindin, Erk 1/2, and p-Erk 1/2 on colorectal tumor and paired normal samples from patients, GAPDH was used as a loading control (* *P*<0.05). (C) Western blot analysis (left panel) and quantitative densitometric analysis (right panel) of mindin on tumor and adjacent normal colon tissues from CAC model of WT mice, GAPDH was used as a loading control.

**Fig. S5** (A and B) The quantitative densitometric analysis of CDK6 and Cyclin D3 expression from Mindin overexpression and Mindin knockdown samples in CMT93 and CT26 cells. The experiment was repeated three times. Mean±SD, **P* < 0.05, ****P* < 0.01. (C) The sequencing chromatogram of the mindin gene knockout mouse.
